# Cellular Stress Responses against Coronavirus Infection: A Means of the Innate Antiviral Defense

**DOI:** 10.4014/jmb.2307.07038

**Published:** 2023-09-08

**Authors:** Ji-Seung Yoo

**Affiliations:** School of Life Sciences, BK21 FOUR KNU Creative BioResearch Group, Kyungpook National University, Daegu 41566, Republic of Korea

**Keywords:** Coronavirus, RIG-I-like receptors, toll-like receptors, antiviral stress granule, Interferon, virus immune evasion

## Abstract

Cellular stress responses are crucial for maintaining cellular homeostasis. Stress granules (SGs), activated by eIF2α kinases in response to various stimuli, play a pivotal role in dealing with diverse stress conditions. Viral infection, as one kind of cellular stress, triggers specific cellular programs aimed at overcoming virus-induced stresses. Recent studies have revealed that virus-derived stress responses are tightly linked to the host's antiviral innate immunity. Virus infection-induced SGs act as platforms for antiviral sensors, facilitating the initiation of protective antiviral responses called "antiviral stress granules" (avSGs). However, many viruses, including coronaviruses, have evolved strategies to suppress avSG formation, thereby counteracting the host's immune responses. This review discusses the intricate relationship between cellular stress responses and antiviral innate immunity, with a specific focus on coronaviruses. Furthermore, the diverse mechanisms employed by viruses to counteract avSGs are described.

## Introduction

Stress, an inherent aspect of life, exerts diverse pressures on all living creatures throughout their lifetimes. Prolonged exposure to stressful conditions has been linked to the development of various clinical problems, including mental and physical disorders. Conditions such as anxiety disorders, depression, autoimmune diseases, chronic pain, and certain types of cancer have been associated with prolonged stress [1, 2]. However, timely and appropriate responses to acute stress induced by specific circumstances can also yield beneficial effects. These responses are essential for maintaining life homeostasis through the intricate relationship between stress and living organisms.

At the cellular level, cells constantly undergo diverse stress conditions induced by multiple stimuli such as toxins, heat, ultraviolet (UV) light, oxidative stress, amino acid deprivation, abnormal protein accumulation, osmotic pressure, and pathogen infection. These stimuli promptly activate a multitude of cellular signaling pathways responsible for stress responses, collectively termed integrated stress response (ISR) [3]. Recent studies have identified crucial molecules involved in the ISR, such as heat shock proteins, RNA chaperones, and endoplasmic reticulum (ER) stress-associated proteins that specifically recognize the stimuli and initiate stress-related signal transduction [4-6].

The activation of stress response programs initiates multiple signaling pathways that lead to the expression of specific genes and a global translation arrest, which are crucial for inflammatory responses. Consequently, these cellular responses play a decisive role in determining the cell's fate, either by promoting cellular recovery or triggering programmed cell death [7]. ISR program initiates by the phosphorylation of the eukaryotic translation initiation factor 2 alpha subunit (eIF2α) by stress response-related kinases. So far, four eIF2α kinases have been identified: heme-regulated inhibitor (HRI), general control nonderepressible kinase-2 (GCN2), double-stranded RNA (dsRNA)-dependent protein kinase (PKR), and PKR-like ER kinase (PERK) [8]. These kinases exhibit stimulus-specific recognition abilities, enabling them to initiate the ISR in diverse cellular stress conditions.

Viral infections also serve as robust stimuli, provoking a cascade of cellular signaling pathways within host cells that play a pivotal role in stress responses. This review aims to provide a comprehensive summary of the current understanding of the molecular mechanisms underlying cellular stress responses, with a particular focus on those induced by coronaviruses. Additionally, this review describes the links between the virus-induced stress responses and host antiviral defense mechanisms. Finally, the immune evasion strategies targeting the host stress responses employed by viruses are discussed.

## eIF2a Kinases as a Stress Sensor

**Fig. 1:**
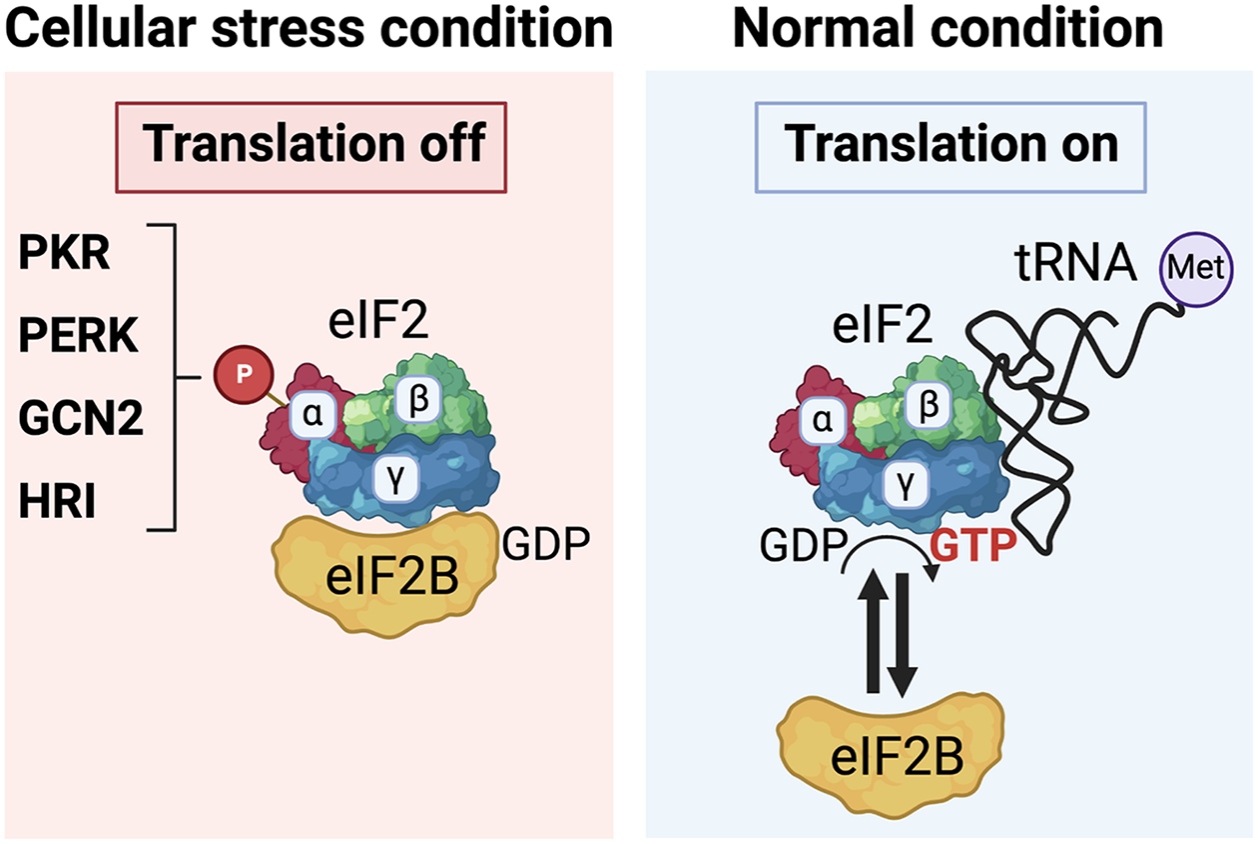
Stress granule formation by four eIF2a kinases. Under normal conditions, the eIF2 complex (composed of eIF2α, eIF2β, and eIF2γ), a crucial molecule that initiates protein translation, functions normally with the assistance of eIF2B, which provides GTP through its guanine-nucleotide exchange activity. However, during stress conditions, eIF2α kinases (PKR, PERK, GCN2, and HRI) detect various cellular stimuli and phosphorylate eIF2α. As a result, the binding affinity between eIF2B and the eIF2 complex increases, causing eIF2B to tightly associate with the eIF2 complex. Consequently, the GTP exchanging function of eIF2B is depleted, leading to the global inhibition of protein translation initiation.

As mentioned above, in response to diverse cellular stress conditions, eukaryotic cells possess a conserved program, termed ISR. ISR can be initiated by activation of one of the four eIF2a kinases, HRI, GCN2, PKR, and PERK. In normal conditions, protein translation is tightly controlled by multiple RNA binding proteins, including translation initiation factors, at the specific compartments where they form a large complex with messenger RNA (mRNA). eIF2 complex, which is composed of three subunits alpha, beta, and gamma, plays a critical role in initiating protein translation. The formation of a GTP-bound eIF2 complex (α, β, and γ subunits) with an initiator methionyl-transfer RNA (Met-tRNA) facilitates the delivery of Met-tRNA to the translation initiation complex. Once transferring Met-tRNA, eIF2g subunit binds to GDP and dissociates from the translation initiation complex. During this regulation, eIF2 requires guanine nucleotide exchanging factor (GEF), eIF2B, as a ‘molecular switch' for the translation on and off [8] (Fig. 1, Normal condition). However, under the cellular stress conditions, eIF2a kinases phosphorylate eIF2a at serine 51. Subsequently, phosphorylated eIF2a tightly binds to eIF2B, and suppresses the GEF function of eIF2B that converts GTP-GDP level at eIF2 complex, leading to halting of the translational machinery, thus arresting a global translation initiation (Fig. 1, Cellular stress condition). By this regulation, cells can avoid unnecessary consumption of energy and nutrition by repressing the protein synthesis during stress conditions. Therefore, eIF2a kinases play a critical role in recognizing cellular stress signals and promoting ISR.

## Involvement of the Stress Sensors in Antiviral Innate Immunity

**Fig. 2:**
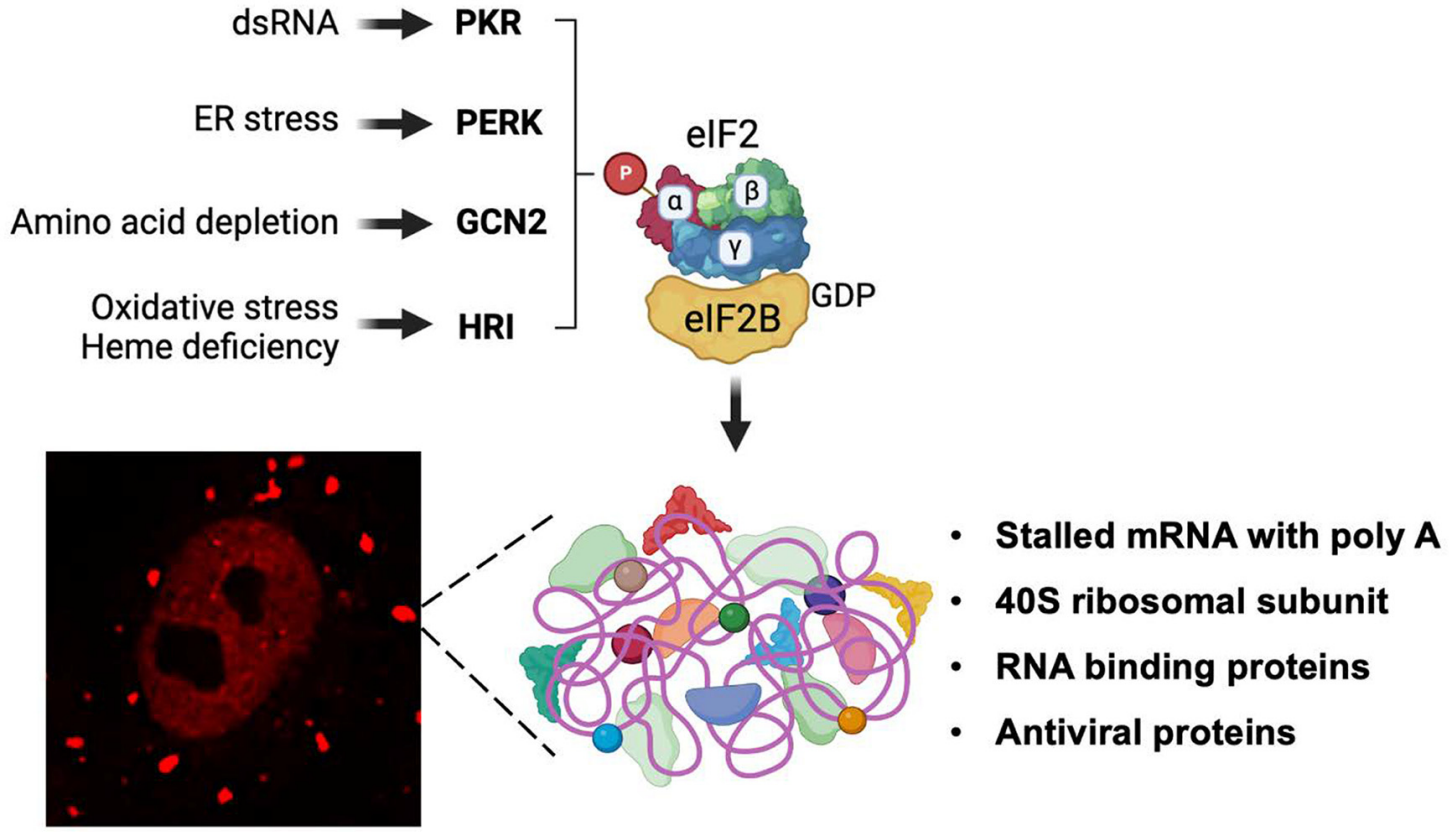
Various stimuli and their specific stress sensor kinases. Different types of stimuli and their respective sensor kinases are depicted. Activation of one of these kinases leads to the prompt formation of stress granules in the cytoplasm, which contain multiple cellular factors.

Interestingly, recent studies have shown that dynamic non-membranous cytoplasmic foci are induced under cellular stress conditions. Researchers have further shown that these granules possess a stalled preinitiation complex that includes 40S ribosomal subunits, RNA binding proteins like translation initiation factors, and polyadenylated mRNAs. Since these foci are present under cellular stress conditions, it was termed “stress granule” (SG) [9]. The key mechanism of SG formation is the phosphorylation of eIF2a by eIF2a kinases. In the following section, the functions of four known eIF2a kinases involved in cellular stress responses, particularly involved in pathogen infection-induced stress conditions, are described (Fig. 2).

### HRI

Although initially identified as a sensor for heme deficiency in erythrocytes, recent studies have revealed that HRI plays a crucial role in regulating various stress responses. Originally, it was discovered that the activation of HRI leads to the phosphorylation of eIF2a and subsequent inhibition of protein synthesis, thereby maintaining a balance in globin levels during erythroid differentiation [10]. Subsequent studies have demonstrated that HRI is also capable of detecting oxidative stress and abnormal protein aggregates within the cytoplasm. McEwen and colleagues demonstrated that HRI is necessary for cell survival under conditions of arsenite-induced oxidative stress [11]. Additionally, it has been reported that HRI controls the accumulation of amyloid-like filament formation of a-synuclein, a critical pathological factor in Parkinson's disease, thus indicating its vital role in sensing cellular proteotoxicity [12].

Recent studies have unveiled an intriguing aspect of HRI's function, revealing its involvement in the regulation of innate immune responses against pathogen infections. Abdel-Nour and colleagues found that HRI is involved in the expression of proinflammatory cytokines, including interleukin-1 (Il1), Il-6, Il-8, and C-X-C chemokine motif ligand 1 (Cxcl1), upon intracellular bacterial infections (such as *Shigella*, *Listeria*, and *Salmonella*), and further elucidated that this regulation occurs through the activation of the ISR pathway [10]. Furthermore, the authors discovered that HRI plays a positive role in inducing type I interferon (IFN) and cytokines upon stimulation of innate immune receptors, such as the retinoic acid-inducible gene I-like receptors (RLRs), toll-like receptor 3 (TLR3), and TLR4. Notably, this regulatory mechanism relies on the mitochondrial antiviral-signaling protein (MAVS) and the TIR-domain-containing adapter-inducing interferon-b (TRIF)-dependent signaling pathway. These findings indicate that the HRI-eIF2a axis selectively regulates innate immune signaling pathways.

### GCN2

GCN2 is an extensively studied cellular stress sensor that detects amino acid depletion within cells. Various pathogen infections often lead to amino acid deprivation as a result of their parasitic nature. For instance, bacterial infections can induce membrane damage, resulting in amino acid depletion [13, 14]. Additionally, viral infections cause amino acid starvation in cells due to vigorous viral replication. An inadequate amino acid supply leads to the accumulation of uncharged tRNAs, which compete with normally functioning charged tRNAs. During amino acid insufficiency, GCN2 recognizes the uncharged form of transfer RNAs (tRNAs) and undergoes a conformational change, resulting in autophosphorylation and activation of GCN2 [15]. Consequently, GCN2 triggers cellular stress responses by phosphorylating eIF2a through its kinase functions.

Recent studies have reported that GCN2-induced stress responses regulate a host antiviral defense program. For example, GCN2 is activated by Sindbis virus (SV) infection through recognition of SV genomic RNA, thereby inhibiting viral translation [16]. Similarly, GCN2 can be activated by viral RNA of human immunodeficiency virus-1 (HIV-1) and exhibits its antiviral activity by inhibiting viral translation [17]. Furthermore, activated GCN2 can suppress HIV-1 replication by interfering with viral integrase function [18]. Interestingly, HIV-1 seems to possess a counter-evading mechanism by cleaving GCN2 protein using viral protease [17]. More recently, it has been demonstrated that GCN2 suppresses dengue virus (DENV) replication by inhibiting the activity of the NF-kB-cyclooxygenase-2 (COX-2) axis pathway. This pathway is essential for various virus replications, including DENV, as it stimulates the production of prostaglandin E2 (PGE2), which exerts a negative modulation on the host immune system [19-21]. This suggests that GCN2 exerts its antiviral activity by regulating the host stress responses.

### PERK

PERK, a well-known sensor of endoplasmic reticulum (ER) stress, is responsible for detecting the accumulation of unfolded proteins in the ER [22]. ER stress commonly occurs during viral infections due to the presence of misfolded viral proteins or disruption of protein processing within the ER. In infected cells, PERK plays a crucial role in restricting viral replication by modulating protein synthesis. When activated, PERK phosphorylates eIF2α, resulting in global inhibition of translation initiation. This inhibition reduces overall protein synthesis in the cell, thereby limiting the production of viral proteins necessary for viral replication [23]. Recent studies have demonstrated that PERK also regulates the inflammatory signaling pathway through the formation of SGs during porcine reproductive and respiratory syndrome virus (PRRSV) infection [24].

Interestingly, several RNA viruses rely on PERK for their replication. For instance, PERK is essential for the translation of nonstructural proteins and the production of negative-stranded viral RNA in the case of the Venezuelan equine encephalitis virus. Furthermore, PERK appears to positively regulate the replication of various flaviviruses, including Rift Valley fever virus and Zika virus, suggesting that PERK plays dual roles, both anti- and pro-viral, in the life cycles of several RNA viruses [25].

### PKR

PKR plays a crucial role in the innate immune response against viral infections by sensing various types of RNA species [26]. Initially identified as a detector of dsRNA [27], recent studies have revealed that PKR can also recognize specific RNA types, such as 5'-ppp with secondary structures, commonly produced during viral infections [28]. Upon recognition, PKR undergoes conformational changes, resulting in homo-dimerization and autophosphorylation. This leads to the phosphorylation of the downstream target protein, eIF2α, which inhibits protein synthesis in infected cells. Consequently, global translation inhibition hinders the production of viral proteins, exhibiting antiviral activity [29, 30].

Furthermore, recent research has demonstrated that PKR contributes to the production of type I IFN [6]. In the context of several viral infections, PKR-mediated SG formation enhances the cytoplasmic sensing pathway for viral RNA. For instance, PKR-mediated SGs are essential for type I IFN production during Newcastle disease virus (NDV), influenza A virus lacking NS1 (IAVdΔNS1), encephalomyocarditis virus (EMCV) infections, or stimulation with synthetic dsRNA, poly(I:C) [31-34]. These findings highlight the multifaceted roles of PKR in antiviral innate immune responses.

## Host Antiviral Responses to Coronavirus Infection

Viruses are infectious agents that elicit multiple cellular stress responses. Upon infection, viruses initiate their life cycle in the host cells by producing viral factors required for replication and propagation. Some viral factors, such as viral genomic DNA, RNA, their intermediate products, and proteins, produced during the virus life cycles can be recognized by host sensors. Sensing of viral invasion by the host innate immune system triggers activation of the programmed pathways for the antiviral defense and stress responses. Activation of the innate immune system initiates by recognizing the specific molecular patterns known as pathogen-associated molecular patterns (PAMPs) through germline-encoded pattern recognition receptors (PRRs). Subsequently, downstream host defense systems get turned on and induce various signal cascades including antiviral interferon signaling pathways to respond to invading harmful factors, such as viruses [35]. In this section, the host strategies of antiviral stress responses against coronaviruses are described.

### Coronavirus

Coronaviruses are a type of enveloped positive-sense single-stranded RNA viruses that belong to the *Orthocoronavirinae* subfamily within the *Coronaviridae* family. The *Orthocoronavirinae* subfamily can be further classified into four genera: *Alphacoronavirus*, *Betacoronavirus*, *Gammacoronavirus*, and *Deltacoronavirus* [36]. Among these viruses, there are seven strains from two genera (*Alphacoronavirus*es and *Betacoronavirus*es) that are pathogenic to humans. Within these strains, HCoV-NL63 and HCoV-229E (*Alphacoronavirus*es) and HCoV-HKU1 and HCoV-OC43 (*Betacoronavirus*es) cause mild respiratory symptoms. However, the other three strains that are transmitted to humans from zoonotic sources, namely SARS-CoV, MERS-CoV, and SARS-CoV-2 (all *Betacoronavirus*es), exhibit unique pathogenesis leading to severe respiratory symptoms and abnormal host antiviral responses [37].

The viral life cycle of SARS-CoV-2 begins with the attachment of the viral spike protein to a specific host receptor called angiotensin-converting enzyme 2 (ACE2), which induces viral fusion with the target cell [38]. After infection, the viral genomic RNA is released from the endosomes, leading to the translation of two open reading frames (ORFs): ORF1a and ORF1b, facilitated by host factors. During translation, two large viral polyproteins, pp1a and pp1ab, are initially synthesized and then further processed into sixteen non-structural proteins (nsp1-nsp16) by two viral proteases: papain-like protease nsp3 and 3C-like protease nsp5. Subsequently, a stable replication complex is formed by viral RNA-dependent RNA polymerase (RdRp) nsp12 and cofactors nsp7 and nsp8, initiating viral replication and transcription. This process generates viral genomic RNA as well as various subgenomic mRNAs. Among the viral proteins produced from the subgenomic mRNAs, ORF3, 6, 7, 8, 9, and 10 modulate various host cellular functions, either benefiting from the host system or evading different cellular innate immune responses. Finally, viral structural proteins such as spike, envelope, membrane, and nucleocapsid are produced, and newly synthesized and matured progeny virions are eventually released through host exocytosis pathways [39].

### Innate Immune Sensors of Coronaviruses

Coronavirus infection triggers a range of immune responses, which involve various viral sensors. Two important types of viral sensors are RIG-I-like receptors (RLRs) [40] and Toll-like receptors (TLRs) [41, 42], which recognize specific RNA species, including viral genomic RNA and replication intermediate RNA. These sensors initiate antiviral signaling cascades. The recognition of viral PAMP RNAs by RLRs and TLRs occurs through distinct mechanisms [37].

**Fig. 3:**
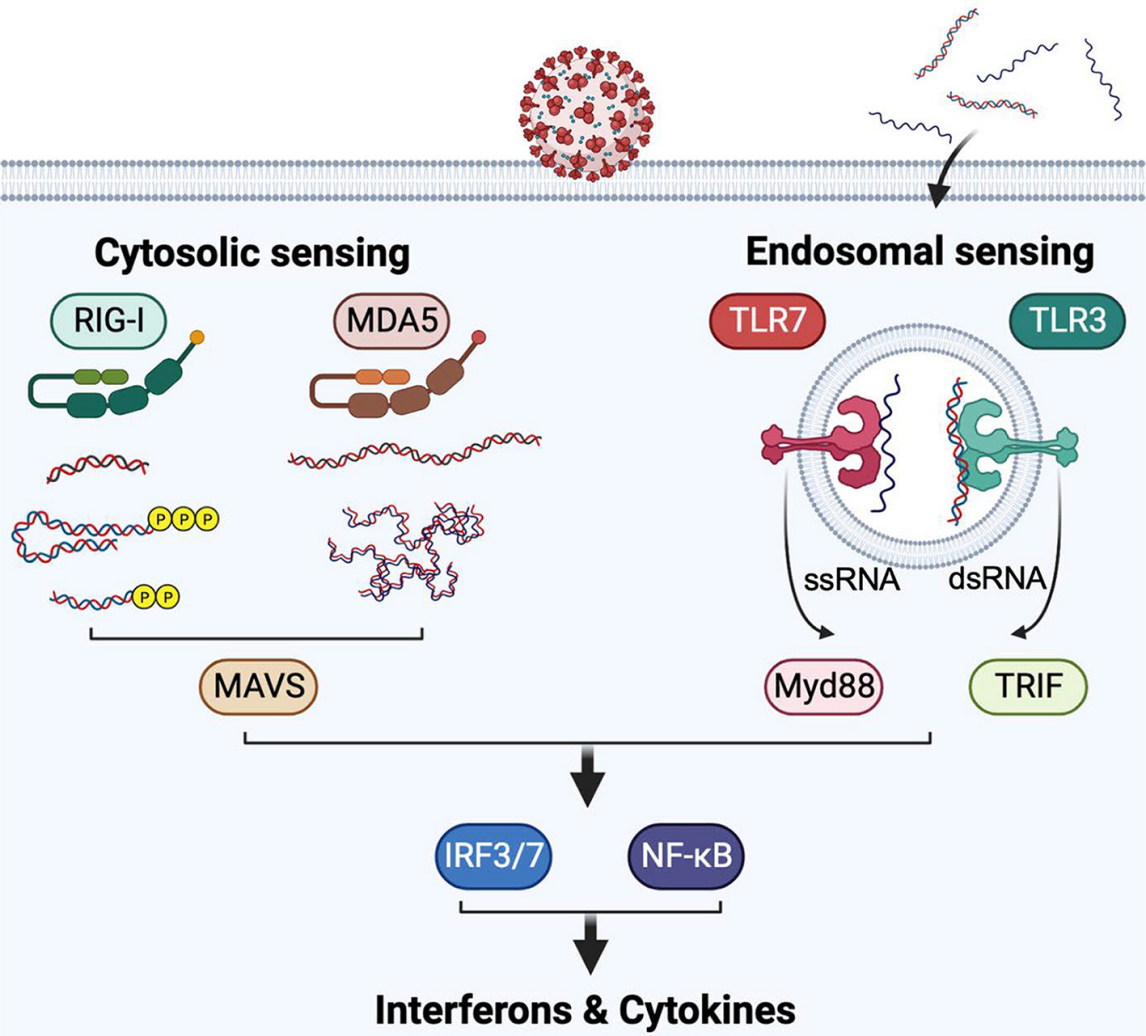
Endosomal and cytoplasmic viral RNA sensing by RLRs and TLRs. Infection of RNA viruses triggers activation of the cytosolic (RIG-I and MDA5) and endosomal (TLR3 and TLR7) RNA sensors. RIG-I and MDA5 detect various types of cytoplasmic viral RNAs, while TLRs recognize endosomal ssRNA (TLR7) or dsRNA (TLR3). Activated RLRs trigger the IFN signaling pathway via interaction with signaling adapter protein, MAVS. Activated TLR3 and TLR7 activate the antiviral IFN program through TRIF and MyD88, respectively, that commonly activate several transcription factors that are involved in the gene expression of antiviral interferons and proinflammatory cytokines.

RLRs detect specific biochemical properties of RNA, such as double-stranded RNA, RNA with a "pan-handle" structure and a 5'-triphosphate group, 5'-diphosphate uncapped RNAs, and RNAs with an unmethylated 5'-end nucleotide at the 2'-O position. Another protein in the RLR family, MDA5, can recognize several species of dsRNA, such as long dsRNA and structurally complex dsRNA, typically produced by viral RNA-dependent RNA polymerase during viral replication [43]. On the other hand, Toll-like receptors (TLRs) responsible for RNA sensing, such as TLR3 and TLR7, localized in endosomes, detect single-stranded RNA (ssRNA) species (TLR7) or dsRNA species (TLR3) associated with viral invasion. Since various coronaviruses produce these RNA species during their life cycle, RLRs and TLRs serve as the primary sensors for coronavirus invasion [35, 44] (Fig. 3).

MDA5 appears to be a predominant sensor for several coronaviruses, including mouse hepatitis virus (MHV) and HCoV-229E infection [45, 46]. MDA5 also collaborates with other RLR family proteins, such as RIG-I and LGP2, in recognizing several coronaviruses. In the case of MHV and MERS-CoV infection, both RIG-I and MDA5 are involved in recognizing the virus and inducing proinflammatory responses [47, 48]. MDA5 and LGP2 are responsible for sensing SARS-CoV-2 and regulating interferon production in lung epithelial cells [49].

Recent studies have highlighted the crucial role of several TLR signaling pathways in the recognition of coronavirus infections. It has been reported that innate immune signaling pathways mediated by TLR3 and TLR4 contribute to antiviral activity against MHV and SARS-CoV [50, 51]. Additionally, TLR7 regulates the production of type I and type III interferons during MHV, SARS-CoV, and MERS-CoV infections [37]. Moreover, recent research has demonstrated that inborn errors or genetic mutations in TLR3 and TLR7 genes result in the failure of antiviral innate immune responses against SARS-CoV-2, leading to severe clinical symptoms [52, 53]. These findings underscore the critical role of TLR-mediated viral sensing in the host defense against coronavirus infections.

## Host Stress Responses as Antiviral Innate Immunity

### Antiviral Stress Granules (avSGs)

**Fig. 4:**
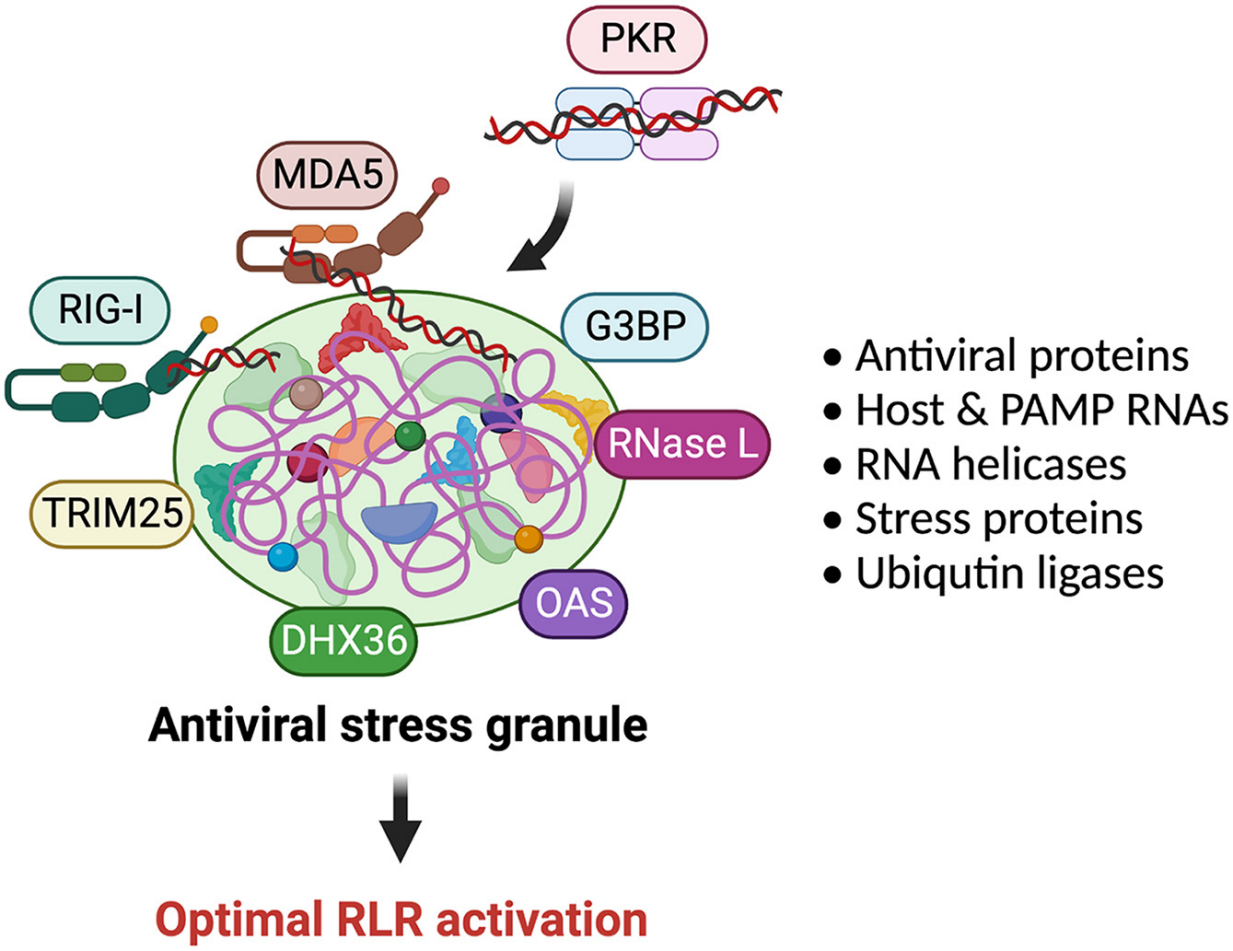
Multiple antiviral proteins are colocalized in the avSGs. Detection of viral PAMP RNAs by cytoplasmic antiviral protein PKR triggers the assembly of stress granules. These stress granules serve as distinct compartments where various host antiviral proteins, stress response-related proteins, and PAMP RNAs are colocalized. The interactions of these molecules within the stress granule facilitate an optimized RLR activation, leading to amplifying the antiviral innate immune responses.

Recent studies have revealed a strong connection between host stress responses and the activation of antiviral sensing programs [54]. As previously discussed, when viral RNA species are detected, PKR promptly initiates cellular stress responses. Onomoto and colleagues made an interesting discovery that SGs, formed through the PKR-eIF2a axis pathway, play a crucial role in the antiviral interferon signaling pathways [31-34]. The researchers observed the colocalization of various antiviral proteins, including RIG-I, MDA5, PKR, OAS, RNAse L, and multiple RNA helicases, within these SGs alongside viral RNAs. Consequently, these antiviral sensor proteins utilize SGs as a "platform" to efficiently detect their ligands (viral RNA species) and interact with other antiviral proteins, and these PAMP RNAs and antiviral aggregates are surrounded by and interact with MAVS [33], a critical adapter molecule of IFN signal transduction, resulting in enhanced and optimized antiviral responses [35]. Conversely, when the host stress responses were inhibited by targeting the PKR-eIF2a axis pathway, the antiviral interferon signaling pathways were nullified. Given these findings, the authors coined this phenomenon as "antiviral stress granule (avSG)" [31, 35] (Fig. 4).

### Virus Immune Evasion Strategies Targeting avSG

Viruses have developed diverse strategies to evade the host antiviral immune system, particularly by targeting the production of interferons derived from avSG. One of the main targets for many viruses is PKR, which plays a crucial role in inducing avSG formation. For instance, influenza virus NS1 protein, Sendai virus C and V protein, and measles virus C protein can suppress avSG formation by inhibiting PKR activation [6].

**Fig. 5:**
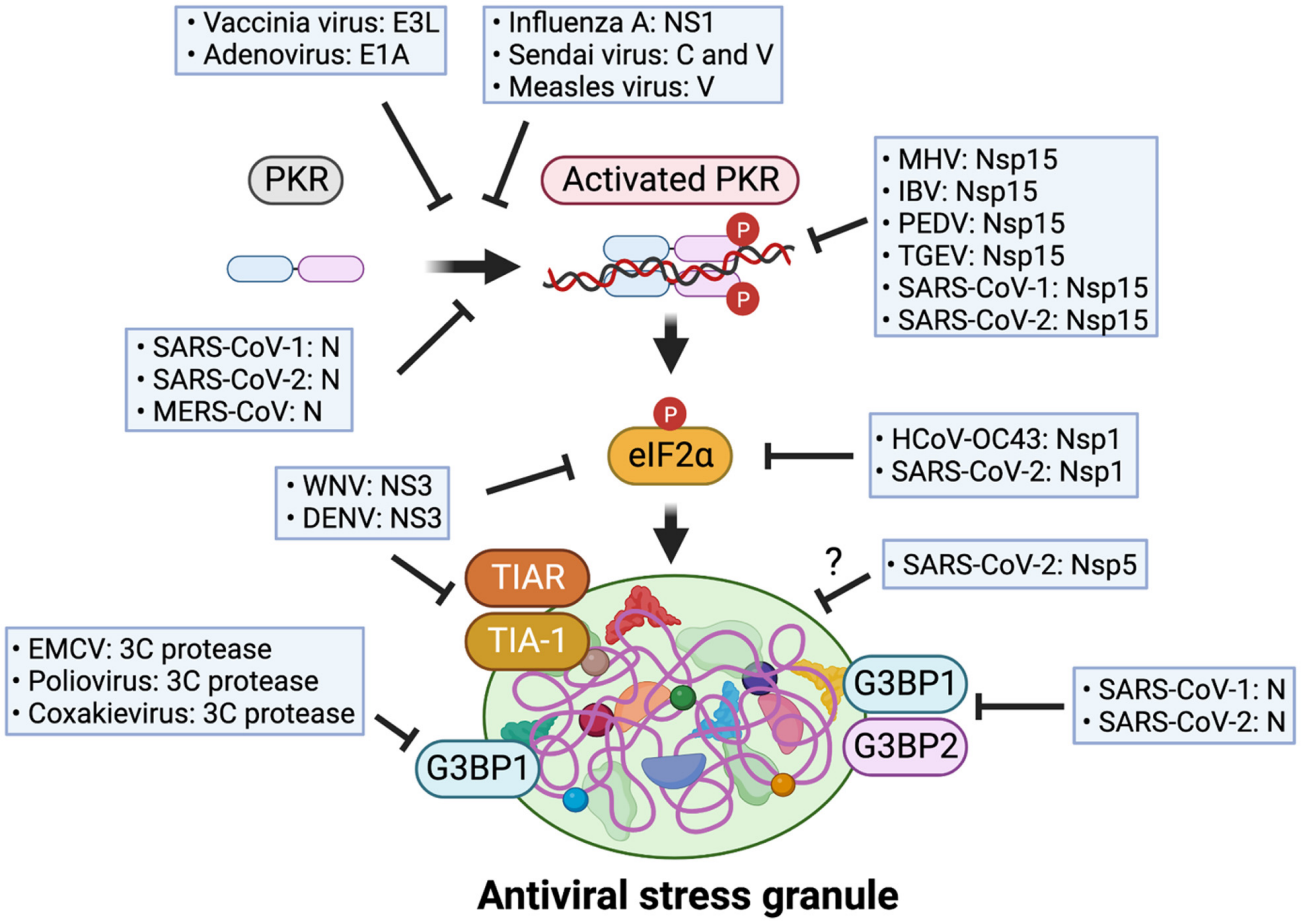
Diverse immune evasion strategies of viruses by targeting avSG formation. Many viruses use specific strategies to evade the host's antiviral program. PKR-eIF2a-avSG axis is targeted by multiple viral proteins that are produced during viral replication, and prevent avSG formation, thereby suppressing innate immune responses.

Additionally, certain viruses target G3BP1 and G3BP2, key factors involved in nucleating SGs, to disrupt the induction of avSG. For example, several *picornaviridae* viruses like EMCV, poliovirus, and coxsakievirus utilize their viral protease, 3C protease, and cleave G3BP1 [34] [55]. Flaviviruses, including West Nile virus (WNV) and Dengue virus (DENV), prevent avSG formation by either targeting SG component proteins, TIA-1/TIAR, or inhibiting the accumulation of eIF2a phosphorylation [56, 57]. Furthermore, DNA viruses like adenovirus and vaccinia virus can suppress interferon production by inhibiting avSG formation through viral proteins E1A and E3L [34, 58]. These findings represent that modulating the host stress responses during viral infection represents a crucial determinant for the survival of both the host and the virus (Fig. 5).

## Immune Evasion of Coronavirus by Targeting avSG

As mentioned, above, virus infections can initiate the formation of SGs through the activation of eIF2α kinases by viral RNAs or other components produced during the viral life cycle. Notably, various coronaviruses generate double-stranded RNA as replication intermediates [39], which prompts the induction of SGs through PKR. Nevertheless, certain coronaviruses, such as SARS-CoV-2, exhibit a robust suppression of SG formation through multiple mechanisms, effectively inhibiting the host's innate antiviral responses. In this section, the strategies employed by coronaviruses to evade the immune system by targeting avSGs, are described (Fig. 5).

### Suppression of avSG Formation by Perturbating SG Inducing Factors

SARS-CoV-2 has demonstrated a remarkable ability to suppress innate antiviral immune responses [37, 59]. One of the mechanisms responsible for viral immune evasion involves the SARS-CoV-2 nucleocapsid (N), which disrupts the formation of avSG. Recent studies found that N protein of SARS-CoV-1, SARS-CoV-2, and MERS-CoV binds to PKR and prevents PKR phosphorylation, thereby inhibiting the formation of avSG [60, 61]. Moreover, N protein of SARS-CoV-1 and SARS-CoV-2 hinder SG formation by targeting Ras-GTPase-activating protein (SH3 domain)-binding protein (G3BP)1 and G3BP2, a key scaffold protein of SG [61-63]. Mechanistically, SARS-CoV-2 N directly binds to the AU-rich region within the 3’ UTR of host mRNAs, effectively sequestering the specific mRNA site where G3BP binds, leading to the attenuation of SG formation [62]. Moreover, Dolliver and colleagues reported that Nsp1 of HCoV-OC43 and SARS-CoV-2 prevent avSG formation by inhibiting eIF2a phosphorylation [64]. Furthermore, SARS-CoV-2 Nsp5, a viral 3CL protease, disrupts the assembly of avSG in a protease activity independent manner [60]. However, the precise underlying mechanism for this inhibitory function of Nsp5 remains elusive.

### Prevention of avSG Formation by Hindering the Accumulation of Viral dsRNAs

Since dsRNA is a primary ligand of virus sensors, sequestration or elimination of viral dsRNA through specific viral proteins can be an efficient way to impede avSG formation. Coronaviruses take advantage of this strategy using Nsp15, which exhibits endoribonuclease function. Deng and colleagues reported that mouse hepatitis virus (MHV) Nsp15 is required for suppressing activation of dsRNA sensors including PKR and MDA5, thereby preventing virus-induce avSG formation [65]. Similarly, recent study showed that several coronaviruses, including infectious bronchitis virus (IBV), porcine epidemic diarrhea virus (PEDV), transmissible gastroenteritis virus (TGEV), SARS-CoV-1, and SARS-CoV-2 impede avSG formation via their Nsp15 by inhibiting accumulation of viral dsRNA [66, 67].

## Perspectives

Stress responses play a pivotal role in maintaining cellular homeostasis in living organisms. Eukaryotic cells possess superior cellular programs that effectively recognize stressful circumstances and promptly initiate protective responses to counteract the stimuli. One such cellular stress response involves the formation of cytoplasmic SGs, induced by eIF2a kinases, which govern protective cellular processes.

Accumulating evidence clearly shows that virus infection-induced SG formation tightly links to the host's antiviral innate immune responses. Given that multiple antiviral proteins, including viral RNA sensors, are localized in the SGs, antiviral signaling pathways are amplified through these avSGs. For viruses, avSGs represent an attractive target to evade the host's antiviral immune response. Indeed, many viruses, including coronaviruses employ diverse strategies to disrupt the formation of avSGs as described above.

Researchers have made substantial progress in investigating virus-induced SG and their critical involvement in antiviral innate immunity. Nonetheless, several essential questions are still unanswered. As SGs are cellular compartments with a large number of proteins that lack a membrane and exhibit dynamic behavior, obtaining a pure SG compartment through purification is technically challenging. Consequently, the precise composition (especially protein types and numbers) of ‘virus infection-specific’ SGs, comprising multiple proteins, remains elusive. Nevertheless, researchers are actively exploring various approaches to overcome these technical difficulties and gain insight into the physiological roles of SGs [68]. A thorough understanding of the mechanism underlying avSG-derived innate immune responses could offer valuable insights to precisely modulate antiviral immunity, which may allow us to apply for designing antiviral therapeutic.
